# Differential expression of miRNA199b-5p as a novel biomarker for sporadic and hereditary parathyroid tumors

**DOI:** 10.1038/s41598-018-30484-9

**Published:** 2018-08-13

**Authors:** Sena Hwang, Jong Ju Jeong, Se Hoon Kim, Yoon Jung Chung, Sun Yong Song, Yang Jong Lee, Yumie Rhee

**Affiliations:** 1 Department of Internal Medicine, CHA University School of Medicine, Chaum Life Center, Seoul, Korea; 2 Department of Surgery, Yonsei University College of Medicine, Seoul, Korea; 3 Department of Pathology, Yonsei University College of Medicine, Seoul, Korea; 4 Brain Korea 21 Project for Medical Science, Yonsei University College of Medicine, Seoul, Korea; 5 Endocrinology, Institute of Endocrine Research, Yonsei University College of Medicine, Seoul, Korea; 6 Department of Internal Medicine, Yonsei University College of Medicine, Seoul, Korea

## Abstract

MicroRNAs (miRNAs) are dysregulated in many tumors; however, miRNA regulation in parathyroid tumors remains poorly understood. To identify differentially expressed miRNAs between sporadic and hereditary parathyroid tumors and to analyze their correlation with clinicopathological features, a microarray containing 887 miRNAs was performed; then, the differentially expressed miRNAs were validated by qRT-PCR using 25 sporadic and 12 hereditary parathyroid tumors and 24 normal parathyroid tissue samples. A receiver operating characteristic curve (ROC) analysis was applied to evaluate the utility of the miRNAs for distinguishing parathyroid tumor types. Compared to the miRNAs in the normal parathyroid tissues, 10 miRNAs were differentially expressed between the sporadic and hereditary parathyroid tumors. Seven of these miRNAs (let-7i, miR-365, miR-125a-3p, miR-125a-5p, miR-142-3p, miR-193b, and miR-199b-5p) were validated in the parathyroid tumor samples. Among these miRNAs, only miR-199b-5p was differentially expressed (*P* < 0.001); miR-199b-5p was significantly downregulated and negatively associated with PTH levels (γ = −0.579, *P* = 0.002) in the sporadic tumors but was upregulated in the hereditary tumors. This miRNA showed 67% sensitivity and 100% specificity for distinguishing sporadic and hereditary parathyroid tumors. These results reveal altered expression of a miRNA between sporadic and hereditary parathyroid tumors and the potential role of miR-199b-5p as a novel biomarker for distinguishing these two types of parathyroid tumors.

## Introduction

Primary hyperparathyroidism (PHPT) is a relatively common endocrine disease with a prevalence of three per one thousand in the general population^1^. PHPT occurs sporadically in up to 90% of cases but may also be a major component of familial syndromes, such as multiple endocrine neoplasia type 1 (MEN1)^2^. MEN1 is an autosomal dominant inherited disease characterized by the occurrence of several endocrine tumors, particularly in the parathyroid gland, endocrine pancreas and pituitary gland^3^. PHPT shows the highest penetrant expression in this syndrome; PHPT occurs in almost 100% of MEN1 patients by the age of 50 yrs, while the MEN1 frequency in PHPT patients is estimated to be 1–18%^4^.

Clinical features, such as age of onset, sex ratio, severity of bone involvement and recurrence rates after parathyroidectomy, are different between sporadic and hereditary parathyroid tumors^5,6^. The discrimination of these tumor types is important because the treatment and disease courses are quite different^7,8^. However, most of the current studies have analyzed the PHPT clinical data without distinguishing the different etiologies^6,9^. Theoretically, PHPT in MEN1 patients is present with multinodular hyperplasia of the parathyroid glands; in contrast, parathyroid adenoma is present in sporadic PHPT. However, the histopathological discrimination between sporadic and hereditary parathyroid tumors is difficult due to a lack of specific abnormalities^10^.

miRNAs are small non-coding RNAs with roles in a wide range of cellular processes in tumorigenesis^11,12^. Currently, many miRNAs have been demonstrated to be diagnostic and prognostic biomarkers for multiple cancer types^13–16^. The potential uses of miRNAs for the effective diagnosis and optimal treatment of parathyroid tumors have also been investigated^17–20^. However, there is little data on parathyroid tumor miRNAs that are differentially expressed between the sporadic and hereditary forms. The purpose of the present study was to identify and analyze differentially expressed miRNAs and to determine their correlation with the clinicopathological features of sporadic and hereditary parathyroid tumors. We determined whether miRNA profiling could serve as a potential biomarker for distinguishing these tumor types.

## Results

### Differences in clinical manifestations between sporadic and hereditary parathyroid tumors

We compared the clinical and biochemical parameters of sporadic and MEN1 parathyroid tumors. The laboratory results showed higher PTH and calcium levels in patients with parathyroid tumors than in normal controls. Patients with sporadic parathyroid tumors had larger tumor sizes and higher levels of PTH and calcium than patients with hereditary parathyroid tumors (Table 1). Consistent with a previous report on the correlation between PTH and tumor volume^21,22^, PTH levels were correlated significantly with tumor size in patients with sporadic parathyroid tumors (γ = 0.592, *P* = 0.002) but not significantly in patients with hereditary parathyroid tumors.

**Table 1:** Clinical and biochemical characteristics.

	Normal parathyroid tissues	Sporadic parathyroid tumors	Hereditary parathyroid tumors
Patient No.	24	25	12
Age	51.2 ± 10.1	54.8 ± 12.4	48.1 ± 12.1
Sex (M:F)	6:18	10:15	4:8
PTH (pg/mL)	31.5 ± 11.0	238.3 ± 178.9*^†^	131.3 ± 73.1
Ca (mg/dL)	9.2 ± 0.5	11.6 ± 0.9*^†^	10.7 ± 1.0^*^
P (mg/dL)	3.7 ± 0.6	2.6 ± 0.3*	2.7 ± 0.4^*^
Cr (mg/dL)	0.7 ± 0.2	0.8 ± 0.2	0.8 ± 0.1
Tumor size (cm)	—	2.0 ± 1.0	1.7 ± 1.0

The data are presented as the means ± SD. One-way between-groups ANOVA with Tukey’s post hoc test. *P < 0.05 *vs*. normal; ^†^*P* < 0.05 sporadic *vs*. hereditary parathyroid tumors.

### Differential expression of miRNA between sporadic and MEN1 parathyroid tumors

**Figure 1:**
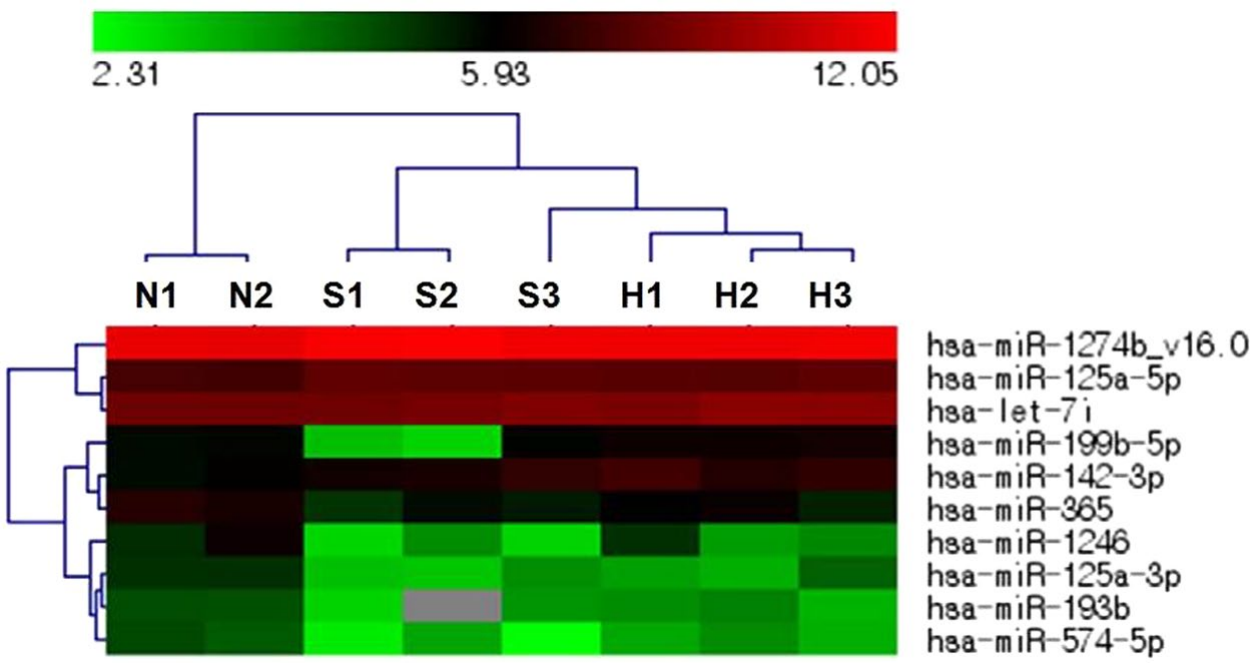
Supervised cluster analysis of miRNA levels in parathyroid tumors. N, normal parathyroid tissue; S, sporadic parathyroid tumor; H, hereditary parathyroid tumor. The data normalized to RNU6 were hierarchically clustered. Red indicates an *increase* relative to all data in this set, and green indicates a *decrease* relative to all data in this set.

A microarray-based supervised cluster analysis for 10 differentially expressed miRNAs in sporadic and hereditary parathyroid tumors versus normal parathyroid tissues is shown (FDR < 0.05) (Fig. 1). Four miRNAs, including miR-365, miR-125a-3p, miR-574-5p, and miR-1246, were significantly downregulated in sporadic parathyroid tumors, whereas miR-142-3p, let-7i, miR-125a-5p, miR-199b-5p, and miR-1274b_v16.0 were significantly upregulated; miR-193b was downregulated in MEN1 parathyroid tumors.

**Figure 2:**
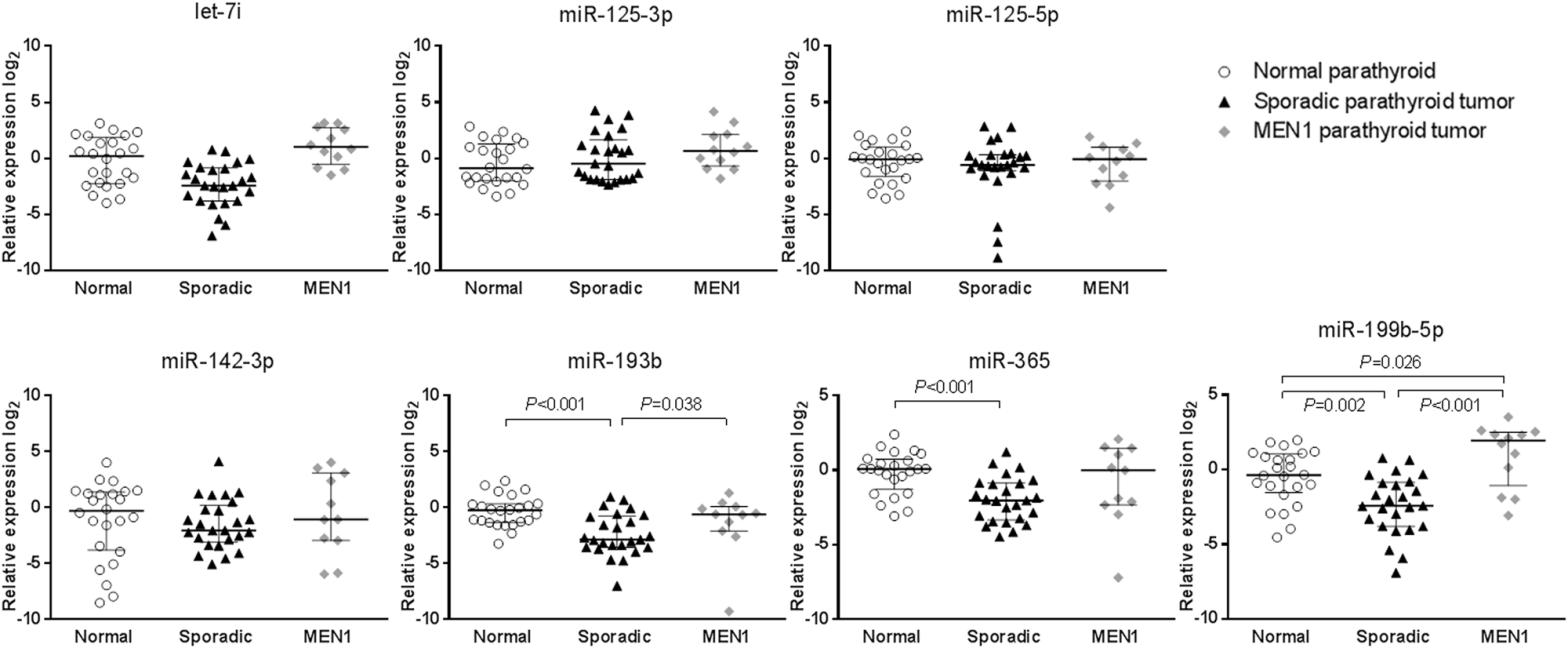
Validation of most relevant miRNAs by qRT-PCR in parathyroid tumors. Scatterplots show relative expression levels of let-7i, miR-365, miR-125a-3p, miR-125a-5p, miR-142-3p, miR-193b, and miR-199b-5p in 24 normal parathyroid tissues, 25 sporadic, and 12 MEN1 parathyroid tumor samples. Horizontal bars represent the median and interquartile range. *P* values were calculated using the Mann-Whitney *U*-test.

Next, seven commercially available miRNAs were used to validate the results in sporadic and MEN1 parathyroid tumors compared with normal parathyroid tissues using quantitative real-time PCR (qRT-PCR) (Fig. 2). The expression levels of miR-193b and miR-365 were lower in sporadic parathyroid tumors than in normal parathyroid tissues, and miR-193b expression was higher in MEN1 parathyroid tumors than in sporadic tumors. Interestingly, only miR-199b-5p had significantly different expression between the two parathyroid tumor types; compared with that in normal parathyroid tissue, miR-199b-5p was downregulated in the sporadic form (median fold change of 0.2) and upregulated in the hereditary form (median fold change of 3.9).

### Discriminating value of miR-199b-5p in parathyroid tumors

**Figure 3:**
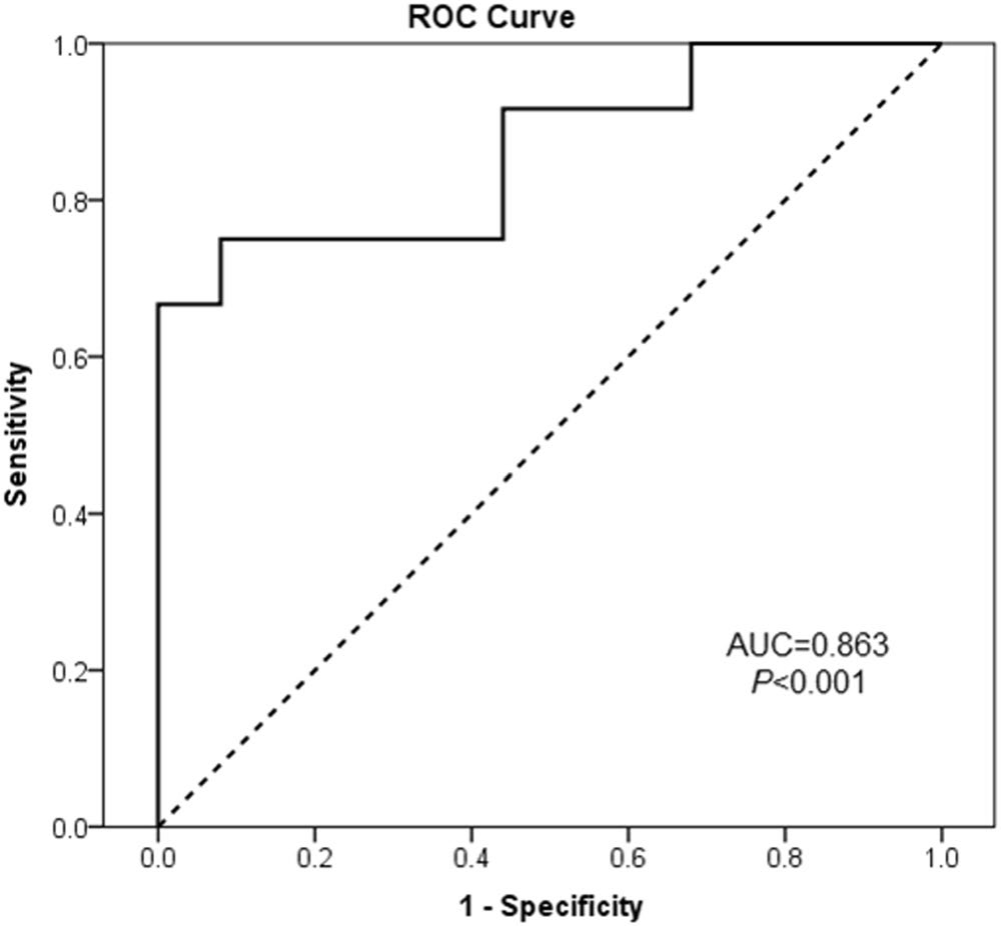
Receiver operator characteristic (ROC) curves of miR-199b-5p showing the discrimination between sporadic and hereditary parathyroid tumors.

The diagnostic relevance of miR-199b-5p was analyzed using a ROC curve analysis (Fig. 3). miR-199b-5p had a large area under the concentration-time curve (AUC = 0.863, *P* < 0.001) with a sensitivity of 67% and a specificity of 100% for discriminating sporadic and hereditary parathyroid tumors.

### Clinical implication of miR-199b-5p in parathyroid tumors

**Figure 4:**
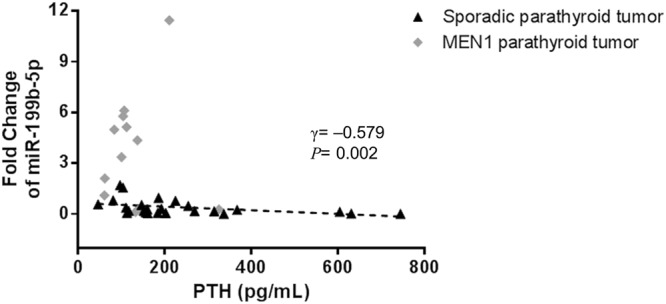
Different correlations between the relative expression of miR-199b-5p and serum PTH levels in parathyroid tumors. A negative association of miR-199b-5p and PTH levels was found in sporadic parathyroid tumors (γ = −0.579, *P* = 0.002), but there was no significant correlation for the hereditary parathyroid tumors.

Given that miR-199b-5p was differentially expressed according to the parathyroid tumor type, we further analyzed the correlation between miR-199b-5p and PTH levels. Interestingly, different correlations between miR-199b-5p and PTH levels in sporadic and hereditary parathyroid tumors were identified: there was a negative association in the sporadic form (γ = −0.579, *P* = 0.002) and no significant correlation in the hereditary form (Fig. 4).

### miRNA target genes and biological function analysis

To inspect the function of miR-199b-5p in parathyroid tumorigenesis, we collected 113 validated miR-199b-5p targets based on miRWalk and miRTarBase (data not shown). Gene ontology (GO) analysis of the candidate target genes showed that DNA-templated regulation of transcription, regulation of cell growth, angiogenesis, regulation of transcription from RNA polymerase II promoter, regulation of cell proliferation, and response to drug are the most significantly enriched GO terms (Table 2). Moreover, KEGG pathway enrichment analysis revealed that the miRNA-targets were significantly associated with pathways in cancer, focal adhesion, and central carbon metabolism in cancer (Table 3).

**Table 2:** GO functional annotation for validated targets of miR-199b-5p according to biological process.

GO Term	Count	%	*P*-value	Genes
GO:0006351 Transcription, DNA-templated	35	31.2	3.0E-8	*DDX3X, E2F3, NAA15, NAB2, POLR2F, ZFP1, CSNK2A1, CCNL1, ERBB2, HES1, HIF1A, MAP3K9, NLK, PAX8, SIRT1, TFDP2, ZBTB37, ZNF117, ZNF195, ZNF215, ZNF286B, ZNF394, ZNF415, ZNF440, ZNF468, ZNF525, ZNF544, ZNF584, ZNF611, ZNF625, ZNF669, ZNF772, ZNF791, ZNF844, ZNF846*
GO:0006355 Regulation of transcription, DNA-templated	29	25.9	1.8E-7	*AKAP17A, SETD2, TSC22D1, ZFP1, HES1, HIF1A, MAP3K9, NLK PAX8, ZBTB37, ZNF117, ZNF195, ZNF215, ZNF286B, ZNF394, ZNF415, ZNF440, ZNF468, ZNF525, ZNF544, ZNF584, ZNF611, ZNF625, ZNF669, ZNF772, ZNF791, ZNF844, ZNF846*
GO:0030307 Positive regulation of cell growth	5	4.5	2.1E-3	*DDX3X TAF9B, CSNK2A1, ERBB2, EXTL3*
GO:0001525 Angiogenesis	7	6.2	3.2E-3	*NAA15, SETD2, HIF1A, JAG1, PLXND1, SIRT1, VAV3*
GO:0045944 Positive regulation of transcription from RNA polymerase II promoter	14	12.5	1.0E-2	*DDX3X, GATA6, JUNB, TAF9B, CCNL1, CDK9, HES1, HIF1A, JAG1, LIF, PAX8, PIN1, SIRT1, TFDP2*
GO:0008284 Positive regulation of cell proliferation	9	8.0	1.0E-2	*CHRFAM7A, E2F3, KIT, CSNK2A1, DYNAP, HES1, KAMC2, LIF, SIRT1*
GO:0042493 Response to drug	7	6.2	1.4E-2	*ABCC1, GATA6, JUNB, CDK9, ITGA3, SLC8A1, VAV3*

**Table 3:** KEGG pathway for most significantly associated targets of miR-199b-5p.

KEGG pathway	Count	%	*P*-value	Genes
Pathways in cancer	7	6.5	0.019	*E2F3, KIT, ERBB2, HIF1A, ITGA3, KAMC2, PAX8*
Focal adhesion	5	4.5	0.026	*ERBB2, ITGA3, LAMC2, VASP, VAV3*
Central carbon metabolism in cancer	3	2.7	0.048	*KIT, ERBB2, HIF1A*

## Discussion

The differential diagnosis of sporadic and hereditary parathyroid tumors remains uncertain. To establish a precise differential diagnosis method for these parathyroid tumors, numerous parameters have been investigated from different clinicopathological conditions^23–25^, but the discrimination remains difficult. Even gene array data showed that hereditary parathyroid tumors are clustered with the sporadic form, indicating that these tumors may share a similar genetic pathway of tumorigenesis^26^. Consequently, new biomarkers are needed for the effective discrimination of these tumor types.

In the present study, miR-199b-5p showed good accuracy for distinguishing sporadic and hereditary parathyroid tumors. The differentiation of these tumor types is associated with improved therapeutic outcomes and decreased recurrence rates of hyperparathyroidism, which requires the reevaluation of uncertain prognoses. These results indicate the need for a more in-depth evaluation of miRNAs in parathyroid tumors. We also observed significantly lower expression of miR-199b-5p in sporadic parathyroid tumors, and miR-199b-5p expression was negatively associated with PTH levels, indicating a specific role for this miRNA in parathyroid tumorigenesis.

Little is known about the different molecular mechanisms between sporadic and hereditary parathyroid tumors that could explain the different disease progression profiles and phenotypes. Several responsible genetic germline changes associated with parathyroid tumors in familial syndromes, such as *MEN1* in MEN 1, *RET* in MEN 2 A, and *CDC73/HRPT2* in HPT-JS (hyperparathyroidism-jaw tumor syndrome), have been identified^27,28^. However, these genetic alterations have also been implicated in a subset of sporadic parathyroid tumors. Genetic alterations in the *MEN1* gene have been reported in 20 to 30% of sporadic parathyroid tumors^29^. Therefore, the presence of constitutively mutated *MEN1* alleles is not sufficient to explain the different tumor profiles between sporadic and hereditary parathyroid tumors.

Substantial advances in the study of miRNA involvement in parathyroid tumorigenesis have been achieved in recent years. Differentially expressed miRNAs between parathyroid carcinoma and adenoma were identified, including miR-139, miR-296, miR-222, miR-503 miR-26b, miR-30b, miR-126*, miR-517c, and miR-372^17–19^. This subset of miRNAs was further verified by Hu *et al*.^20^. Emerging evidence has shown that even the partial inactivation of tumor suppressors can importantly contribute to tumorigenesis^11^. In this context, miRNAs can be good candidates for the subtle regulation of gene expression based on a continuum model of tumor suppressor gene function. Interestingly, this presumption was confirmed, in part, by Luzi *et al*., showing that miR-24-1 could bind to the *MEN1* mRNA and inhibit menin expression, closing a feedback loop^30^. Grolmusz *et al*. also reported miR-24 and miR-28 were differentially expressed between sporadic and MEN1 parathyroid tumors^31^. Subsequently, miR-4258, miR-664, and miR-1301 were demonstrated to involve in the MEN1 associated parathyroid tumors^32^. Unfortunately, however, miR-199b-5p was not identified in previous studies. This inconsistent result might be due to small sample size because of rarity of MEN1 related samples and genetic heterogenesis of parathyroid tumors^20^.

Several studies related to miRNAs have shown that miR-199b-5p is a putative tumor suppressor that targets several signaling pathways: Hes1 involved in both Notch and Hedgehog pathways in medulloblastoma^33^ and osteosarcoma^34^, PODXL and DDR1 in acute myeloid leukemia^35^, HIF-1α in hepatocellular carcinoma^36^, and HER2 and its downstream signaling ERK1/2 and AKT pathway in breast cancer^37^. Taken together, the overexpression of miR-199b-5p could significantly inhibit cell proliferation, migration, and clonogenicity. Interestingly, miR-199b-5p could be a fine tuner of target gene expression, suggesting its epigenetic control function during tumor development^33^. Although its exact functions in parathyroid tumors are unknown, miR-199b-5p was found to be negatively correlated with serum PTH which associated with tumor size in sporadic parathyroid tumors in our study. Moreover, GO analysis suggested that miR-199b-5p targeted genes may play roles in transcription regulation, regulation of cell growth and proliferation, and angiogenesis. The most significant pathway in KEGG analysis was pathways in cancer. Altogether suggests that miR-199b-5p could play a possible role in parathyroid tumorigenesis.

**Figure 5:**
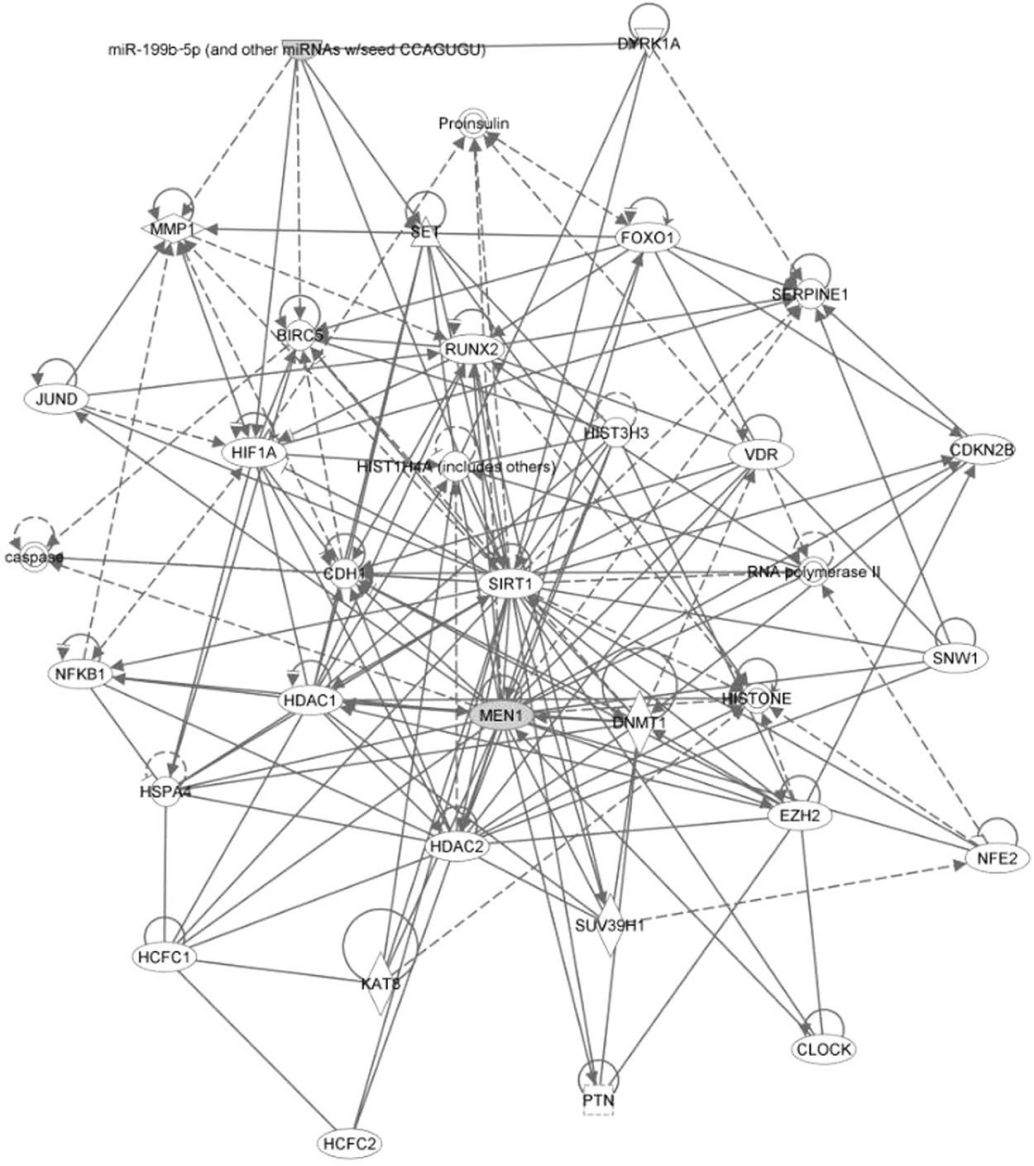
A network predicted to be regulated by miRNA-199b-5p and the *MEN1* gene. One predicted network regulated by miRNA-199b-5p and the *MEN1* gene was “Gene expression, cellular development, cellular growth and proliferation”.

A significant direct association between miR-199b-5p and PTH levels in MEN1 parathyroid tumors was not observed in the present study, which may be due to the complex interactions between genetic backgrounds and other susceptibility factors affected by the *MEN1* gene. To investigate the potential effects of miR-199b-5p on parathyroid tumorigenesis in different genetic backgrounds, we used bioinformatics to predict a network between miR-199b-5p and the *MEN1* gene with Ingenuity Pathway Analysis (IPA) software (Ingenuity® Systems version 8.0, www.ingenuity.com) (Fig. 5). Interestingly, one pathway was identified: gene expression, cellular development, cellular growth and proliferation. According to the IPA results, miR-199b-5p directly targets the transcription regulators HIF-1α and SIRT1, which play a key role in promoting cell proliferation^38,39^ and have known interactions with *MEN1*^40^. However, it is unclear how altered miR-199b-5p expression occurs within the context of dysregulated *MEN1* in parathyroid tumors. The ability of miR-199b-5p to mediate the expression of these two genes may also explain the relationship between miR-199b-5p and *MEN1*. These complex relations must be confirmed in further investigations. There are some limitations to this study. First, the sample size of MEN1-related parathyroid tumors is small due to the rarity of this condition. Second, the lack of experimental validation for functional studies of miR-199b-5p and its predicted target genes is a further limitation.

In conclusion, we identified that miR-199b-5p is differentially expressed between sporadic and MEN1 parathyroid tumors and could be a potential diagnostic marker for distinguishing these tumors in different genetic backgrounds. Considering these data on the association between PTH and miR-199b-5p in parathyroid tumors, it will be important to focus future studies on the role of miR-199b-5p in parathyroid tumorigenesis.

## Materials and Methods

### Parathyroid tissue samples

We obtained a total of 61 parathyroid tissue samples and the associated clinical and histopathological data. Thirty-seven parathyroid tumor tissues were obtained from parathyroidectomy procedures. Twelve samples were obtained from MEN1 patients and confirmed to have germline mutations in the MEN1 gene. Twenty-four normal parathyroid tissues were used as controls; these tissues were incidentally removed during thyroidectomy in hyperthyroidism patients who had no evidence of PHPT. The present study was approved by the Institutional Review Board of Severance Hospital (4-2011-0613), and written informed consent was obtained from all patients. All experiments were performed in accordance with the relevant guidelines and regulations.

### RNA isolation

Total RNA was extracted from the formalin-fixed and paraffin-embedded (FFPE) samples using TRIzol reagent (GIBCO, BRL, Gaithersburg, MD, USA) according to the manufacturer’s protocol. Following extraction, total RNA was quantified by an ND-1000 spectrophotometer (NanoDrop Technologies, Rockland, DE, USA).

### MicroRNA microarray

For the miRNA microarray study, 3 sporadic and 3 MEN1-related parathyroid tumors and 2 normal parathyroid tissue samples were used. The quantitation of mature miRNA expression levels in parathyroid tissues was performed using a human miRNA Microarray Release 14.0, 8 × 15 K (Agilent, Waldbronn, Germany), which contains 887 human miRNAs with four duplicate probes per miRNA. The hybridization signals were detected by an Agilent SureScan microarray scanner. The scanner images were analyzed by Agilent feature extraction software. Data normalization was performed with Genowiz 4.0.5.6. An adjusted *P*-value controlling for a false discovery rate (FDR) of < 0.05 was used to identify miRNAs that were differentially expressed between parathyroid tumors and normal parathyroid tissues.

### Quantitative real-time PCR

miRNAs were validated in 25 sporadic and 12 hereditary parathyroid tumors and 24 normal parathyroid tissues by qRT-PCR. The levels of mature hsa-let 7i, miR-125a-3p, miR-125a-5p, miR-142-3p, miR-193b, miR-199b-5p, and miR-365 were measured using individual TaqMan microRNA assays (Applied Biosystems) according to the manufacturer’s instructions. Total RNA (15 ng/15 µL of reaction) was converted into complementary DNA (cDNA) using a TaqMan miRNA reverse-transcription kit (Applied Biosystems, Carlsbad, CA, USA); then, the cDNA was subjected to amplification with TaqMan Universal PCR Master Mix and an ABI 7500 quantitative PCR machine (Applied Biosystems, Carlsbad, CA, USA). The following commercially available and prevalidated TaqMan® primers/probes for stem-loop miRNA were used: let-7i (002172), hsa-miR-125a-3p (002199), hsa-miR-125a-5p (002198), hsa- miR-142-3p (000464), hsa-miR-193b (002367), 199b-5p (000500), and hsa-miR-365 (001020). The expression miRNA levels in the samples were normalized to RNU6. miRNA expression levels were analyzed for relative fold-changes from the threshold cycle (Ct) values using the 2^−ΔΔCt^ method^38^.

### *In silico* miRNA target prediction and functional analysis

The validated target genes of miRNA were predicted via miRWalk^41^ and miRTarBase^42^. GO term and KEGG pathway analysis were performed for the candidate genes using the DAVID gene annotation tool (http://david.abcc.ncifcrf.gov). The enrichment *P* values of both GO and KEGG pathway enrichment analysis were set as significant when *P* < 0.05.

### Statistical analysis

Statistical analyses were performed using SPSS 18.0 software (SPSS, Inc. Chicago, IL, USA). Differences in continuous variables between the three groups were tested by one-way ANOVA with Tukey’s post hoc test, and the differences between two groups were determined by the independent sample *t*-test or Mann-Whitney *U*-test. Spearman’s rank correlation coefficients were used to assess the associations between the relative miRNA expression and PTH levels. Receiver operating characteristic curve (ROC) analysis was applied to obtain the utility of the miRNA for distinguishing between sporadic and hereditary parathyroid tumors. A value of *P* < 0.05 was considered statistically significant.
